# Effectiveness and Utility of Virtual Reality Infection Control Simulation for Children With COVID-19: Quasi-Experimental Study

**DOI:** 10.2196/36707

**Published:** 2022-05-27

**Authors:** Mi Yu, Mi Ran Yang

**Affiliations:** 1 College of Nursing Institute of Health Sciences Gyeongsang National University Jinju Republic of Korea; 2 Department of Nursing Kwangju Health University Kwangju Republic of Korea

**Keywords:** children, infection control, nursing student, simulation training, virtual reality, digital health, medical education, COVID-19, patient management, pediatrics, nursing education, respiratory care skills, program usability, digital learning

## Abstract

**Background:**

It is essential that nurses quickly learn the proper methods for preventing and controlling nosocomial infection and managing intensive care patients during the COVID-19 pandemic, including the donning and doffing of personal protective equipment (PPE). Virtual reality (VR) simulation offers the advantage of learning in a safe environment with a sense of realism similar to that of an actual clinical setting and has been reported to enhance self-efficacy in infection control, safety performance, and learning satisfaction among students.

**Objective:**

This study aims to develop a virtual reality infection control simulation (VRICS) program regarding donning and doffing of PPE and respiratory care for pediatric patients admitted to an isolation unit for COVID-19 and to identify the effects of the program on PPE knowledge, infection control performance, and self-efficacy for nursing students. Additionally, the realism of the VRICS program and the students’ level of satisfaction with the program were assessed.

**Methods:**

This was a quasi-experimental study based on a controlled pretest-posttest design. Third- and fourth-year nursing students were divided into an experimental group (n=25) who participated in a VRICS program and a control group (n=25) with no participation. Data were collected from November 13 to December 10, 2021, and analyzed using descriptive statistics and the *t* test, paired *t* test, Mann-Whitney U test, and Wilcoxon matched-pair signed-rank test. The VRICS program consisted of a prebriefing, including direct practice of donning and doffing PPE, VR simulation, and debriefing. The VR simulation comprised 3 sessions: donning and inspection of PPE in the dressing room before entering the negative-pressure isolation unit; assessing for suction care, nasopharyngeal suctioning, and checking of COVID-19 patients in the negative-pressure isolation unit; and doffing PPE in the dressing room. The total execution time for the program was 180 min.

**Results:**

Compared with the control group, the experimental group showed significantly greater improvements in PPE knowledge (*z*=–3.28, *P*<.001), infection control performance (*t*_48_=4.89, *P*<.001), and self-efficacy (*t*_36.2_=4.93, *P*<.001). The experimental group’s mean scores for realistic immersion and learner satisfaction were 4.49 (SD 0.50) points and 4.75 (SD 0.38) points (on a 5-point Likert scale), respectively.

**Conclusions:**

The VR simulation training program involving pediatric COVID-19 patients combined skills training effectively and enhanced theoretical knowledge, respiratory care skills, and infectious disease preparedness. Thus, it could be applied to training nurses to respond more effectively to public health situations involving infectious diseases, including the COVID-19 pandemic.

## Introduction

Health care professionals, including nurses, who care for COVID-19 patients, are at direct or indirect risk of exposure to the virus or other infectious substances. Failure to comply with the appropriate control guidelines can make them a source of infection or a mediator that can spread COVID-19 [1]. Despite the vital role they play, most clinical nurses believe that they lack experience and knowledge in disaster nursing, such as COVID-19, and rate their personal emergency preparedness low [2]. Thus, it is essential that nurses quickly learn the proper methods, including the donning and doffing of personal protective equipment (PPE) for preventing and controlling nosocomial infection and managing intensive care patients during the COVID-19 pandemic [3]. In line with this, thorough and systematic education and training are needed beginning at the nursing undergraduate level. Nursing students need to experience nursing in actual clinical settings, but they get limited opportunities to personally observe and practice COVID-19-related infection control nursing, as well as acquire sufficient nursing skills. In particular, practicing pediatric nursing, including care of infants, is even more challenging due to the increased vulnerability of the child to infection [4]. Therefore, college instructors need to develop curricula to enable nursing students to cope with infectious diseases. However, a large gathering of students should be avoided in the middle of a pandemic to avoid the risk of viral transmission, and thus, new training methods should be applied. Accordingly, nursing training programs using advanced technologies are being developed [5], including a growing trend in virtual reality (VR) [4,6,7].

VR refers to a technology that creates a simulation of real surroundings with people actually experiencing an environment that may be difficult to experience in daily life [8]. VR simulation-based training deals with virtual patients within VR space instead of actual patients and thus does not threaten the safety of patients, allowing the learners to repeatedly practice comfortably on their own within the virtual space [8]. Moreover, it is a user-centric learning method that does not depend on preparations, personnel, and schedule, while allowing the users to practice various clinical techniques within a safe environment, unlike conventional high-fidelity simulation [9]. A recent meta-analysis [10] and systematic review [11] indicated that the use of VR simulation has the potential to produce educational outcomes similar or superior to those of traditional simulation and reported the positive effects of VR on self-efficacy [4,12] and learning satisfaction [4,12,13] among nursing and medical students. Meanwhile, there are mixed results, too, showing that performance increased in the experimental group [12-14] or there was no difference between groups [15].

Yu et al [4] developed a VR program for high-risk neonatal infection control and applied it to nursing students. The results showed improved confidence in infection control among the students, indicating the applicability of VR simulation as a simulation-based training program for nursing students. The study also reported that VR simulation offers the advantage of learning in a safe environment with a sense of realism similar to that of an actual clinical setting, but without major time constraints, and how the instructor carries out the lesson. Moreover, Birrenbach et al [13] compared an immersive VR simulation with a traditional learning method for a COVID-19-related skill set. This before-and-after training for medical students involved the performance of hand disinfection, nasopharyngeal swabbing, and the donning and doffing of PPE. The results showed that safe performance scores, such as for nasopharyngeal swabs, increased more in the VR simulation group than in the control group. Moreover, VR simulation provided user satisfaction, while remaining as effective as conventional learning methods for medical students. Such findings demonstrated that VR could be a useful tool for acquiring simple and complex clinical skills.

Meanwhile, prebriefing, with its importance in simulation emphasized recently [16], is a structured simulation stage before the scenario stage and is carried out by the instructions of the simulation moderator based on the experience and knowledge of the participants. It also includes activities for preparing how to use equipment or supplies in the simulation processes. It can also be viewed as a stage for creating a safe and reliable learning environment to promote participation and help achieve learning goals [16,17]. Accordingly, this study used prebriefing based on the self-efficacy theory by Bandura [18] and designed the program to enhance self-efficacy [19] through dynamic learning experience by actually performing the actions and proxy experience from observing the actions of others [20]. Individuals who have high self-efficacy will exert effort that, if well-executed, leads to successful performance and outcomes [18], such as clinical competency and simulation performance [21].

However, there are limited VR simulation programs on medical conditions, treatment, and patient care for training nurses or nursing students for responding to respiratory infectious disease epidemics. In particular, infection control in hospitals has become more important due to the COVID-19 pandemic and PPE, such gloves, disposable gowns, N95 masks, protective goggles, and shoe covers, can be a primary physical barrier against infection. Despite this, it is difficult to find virtual reality infection control simulation (VRICS) programs applied to pediatric patients for donning PPE, which is not normally used and may appear unfamiliar and complicated. This study thus aims to develop and test a VR simulation program incorporating pediatric COVID-19 cases.

The objective of this study was to develop a VRICS program regarding donning and doffing of PPE and respiratory care for pediatric patients admitted to an isolation unit for respiratory infectious disease and apply the program to nursing students to identify the effects of the program on PPE knowledge, infection control performance, and self-efficacy, as well as the realism of the VRICS program and the level of satisfaction with the program. Accordingly, the study established the following hypotheses:

- Hypothesis 1: The experimental group (participation in the VRICS program) and control group (no participation) will show a difference in PPE knowledge.
- Hypothesis 2: The experimental and control groups will show a difference in infection control performance.
- Hypothesis 3: The experimental and control groups will show a difference in self-efficacy.

## Methods

### Study Design

This was a quasi-experimental study based on a controlled pretest-posttest design for the development of a VRICS program with the objective of comparing PPE knowledge, infection control performance, and self-efficacy between the participating and the nonparticipating group of nursing students, as well as to identify the level of satisfaction with the program.

### Study Population

The target population was all nursing students in Korea, and the study selected third- and fourth-year nursing students from a nursing college in “J” City in Gyeongsangnam Province. The inclusion criteria consisted of nursing students currently enrolled in a nursing college who have clinical practice experience, completed courses on pediatric nursing and a core basic nursing skill course titled “Practice for Donning and Doffing Standard PPE and Managing Medical Waste” in the regular curriculum, and voluntarily consented to participate in the study. The exclusion criterion was the lack of consent to participate in the study. The sample size for the study was calculated using G*Power version 3.1.9.7 [22]. Considering that the effect size was 0.40-0.72 in previous studies, which is similar to that in this study [23-25], a moderate effect size (f) of 0.50, a significance level (α) of .05, and a statistical power (1 – β) of .90 were used, with a suitable sample size (2-tailed test) of 44. Considering a dropout rate of 10%, a total of 50 participants were recruited for the final study population, with 25 (50%) participants each in the experimental and control groups.

### Study Instruments

#### General Characteristics

The general characteristics of the participants included age, sex, previous semester grades, and VR experience.

#### PPE Knowledge

PPE knowledge was measured using the tool originally developed by Choi [26] for PPE knowledge related to acute respiratory infection, which was modified and supplemented to be suitable for COVID-19. The tool consisted of 20 items: 2 items on the transmission route, 3 items on hand hygiene, and 15 items on donning and doffing PPE. Each correct answer was given 1 point, and a wrong answer was given 0 points. The total score ranged from 0 to 20 points, with higher scores indicating a higher knowledge level. The content validity index of the tool at the time of development was 0.8-1.0 for each item, while the Kuder-Richardson Formula 20 (KR-20) of the knowledge items in the study was 0.68.

#### Infection Control Performance

Infection control performance was measured based on items related to PPE and pediatric respiratory care. PPE-related items were measured using the tool originally developed by Kwon [27] for measuring PPE use among nurses in specialized infectious disease hospitals, which was modified and supplemented according to the actual performance procedures of this study. The tool consisted of 20 items, and each item was rated on a 5-point Likert score. The total score ranged from 20 to 100 points, with higher scores indicating higher performance. The reliability of the tool (Cronbach α) was .86 in the study by Kwon and .97 in this study.

#### Self-efficacy

Self-efficacy refers to the personal belief about whether something new that has been learned can be applied [28]. Self-efficacy was measured using the tool originally developed by Ayres [28] and adapted by Park and Kwon [29] for simulation studies. The tool consisted of 10 items, and each item was rated on a 7-point scale (1=not at all to 7=very much so). The total score ranged from 7 to 70 points, with higher scores indicating higher self-efficacy. The reliability of the tool (Cronbach α) was .94 at the time of development, .95 in the study by Park and Kwon [30], and .94 in this study.

#### Realistic Immersion

Realistic immersion refers to the perception of presence in an environment that provides realistic illusion [30]. Realistic immersion was measured using the items corresponding to realistic immersion within the tool for measuring presence developed by Chung and Yang [31] for 3D video assessment. This study used 3 items: “The appearance of the VR simulation program video seemed to be real,” “While learning the VR simulation program, the screen seemed to exist in reality,” and “I felt like I was participating in a real field while learning the VR simulation program.” Each item was rated on a 5-point Likert scale (1=strongly disagree to 5=strongly agree). The highest possible score was 15 points, with higher scores indicating higher realistic immersion. The reliability of the tool (Cronbach α) was .75 in the study by Chung and Yang [31] and .76 in this study.

#### Satisfaction With the Program

Satisfaction with the VR simulation program was measured after providing the program to the participants using 3 items developed by Yu et al [4] and modified for this study, which were as follows: “This program will help me work as a nurse in clinical practice,” “I want to recommend this program to other nursing students,” and “This training is necessary as part of the nursing college curriculum.” Each item was rated on a 5-point Likert scale (1=strongly disagree to 5=strongly agree). The highest possible score was 15 points, with higher scores indicating higher satisfaction with the program. The reliability of the tool (Cronbach α) was .81 in the study by Yu et al [4] and .81 in this study.

### VRICS Program

The VRICS program for patients with COVID-19 was constructed in the order of prebriefing, including prepractice, VR simulation, and debriefing (Table 1). This program was conducted with 6-7 people participating per session, and a total of 4 sessions operated.

**Table 1:** Program design of the VRICS^a^ (overall time expended=180 min).

Procedure		Contents and situation	Time expended (min)
**Prebriefing session**			
	Introduction of scenarios	Simulation scenarios, theory of respiratory care skills for patients with COVID-19	25
	Proxy and prepractice	Watching the video, practicing donning and doffing PPE^b^	15
	Orientation and precautions for VR^c^	Overview of the VR simulation lab environment, including the use of VR equipment, such as the HMD^d^ and leap motion controller as well as disposable eye masks for the headset to prevent cross-contamination	10
**VR simulation session**			
	Donning of PPE, respiratory care, doffing of PPE	Patient status check and nasal-oral suction care: Patient: 1-year-old infant Diagnosis: R/O COVID-19 Symptom: Lung sound with crackle, heart rate 140 beats/min, oxygen saturation (SpO2) 95% Respiration rate: 20 breaths/min	90-110
**Debriefing session**			
	Discussion	Students reflecting on the simulation experience and exchanging feedback with the instructor	20

^a^VRICS: virtual reality infection control simulation.

^b^PPE: personal protective equipment.

^c^VR: virtual reality.

^d^HMD: head-mounted display.

#### Prebriefing

Prebriefing in this study consisted of standard prebriefing, along with demonstrations and actual practices. The participants received orientation on the goal of the simulation, introduction, review of scenarios, and the simulation lab environment. Subsequently, knowledge transfer learning regarding COVID-19 infection and suction care for preventing droplet transmission and oxygen therapy in a negative-pressure environment for inpatients with COVID-19 was introduced. The participants were instructed to watch an expert role-modeling video on donning and doffing PPE. Each participant practiced donning and doffing PPE for 15 min. During this process, the instructor corrected any errors made by the participants and encouraged the participants to perform the tasks accurately. Moreover, the participants were allowed to take photographs of each other while wearing PPE to experience success. This was to maximize the effects of VR simulation by carrying out learning using the actual experiences of the students. Users unfamiliar with VR technology may need additional time to become familiar with the controller [32]. Therefore, the instructor gave a personal demonstration for 10 min on VR-related equipment (head-mounted display [HMD] and leap motion controller), environment, and supplies in the VR simulation lesson room to enable imitation learning. The participants were allowed to gain proxy experience by watching a computer virtual screen and other people performing the tasks during the VR simulation lessons.

#### VR Simulation

**Figure 1:**
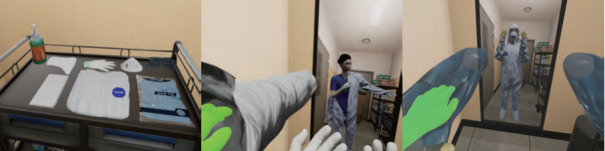
VR simulation session 1: dressing zone (clean area). Checking the PPE, handwashing (HW) > donning PPE (inner gloves > waterproof long-sleeved gown > shoes > N95 mask > goggles > hood > outer gloves) > checking the condition of the PPE by looking in the mirror. PPE: personal protective equipment; VR: virtual reality.

**Figure 2:**
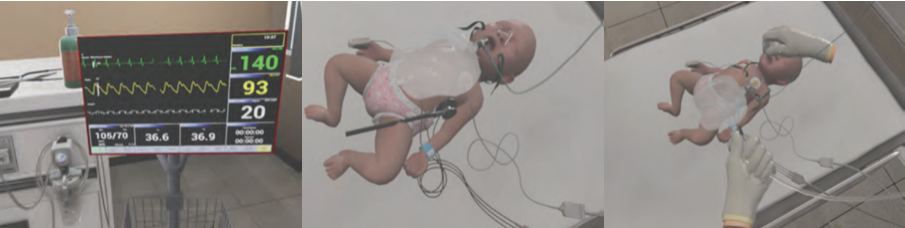
VR simulation session 2: negative-pressure isolation room. Checking patient identification, assessing patient condition > auscultating lung sound > respiratory care (oral and nasal suctioning). VR: virtual reality.

**Figure 3:**
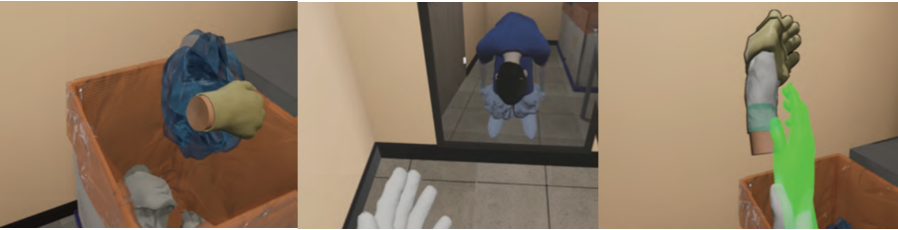
VR simulation session 3: changing zone (contaminated area). Doffing PPE (shoes > handwashing [HW] > outer gloves > HW > gown > HW > goggles > HW > N95 mask > HW > inner gloves > HW). PPE: personal protective equipment; VR: virtual reality.

The VR simulation consisted of 3 sessions: (1) donning and inspection of PPE in the dressing room before entering the negative-pressure isolation room (Figure 1), (2) nasopharyngeal suctioning and assessment for suction care and checking for pediatric patients with COVID-19 in the negative-pressure isolation room (Figure 2), and (3) doffing PPE in the contaminated area (Figure 3).

To operate the VR simulation program, an instructor’s computer, the learner’s HMD, and multimedia learning equipment were set up and a VR learning space with a radius of approximately 3 m from the learner was created so that learner movement and depth could be detected, as well as any danger during such movement. In addition, a user interface (UI) was created to induce movement to subsequent steps or to provide brief guidance on some procedural details. Each scenario was carried out for 15 min per participant. The program was designed to allow students who were not taking part in the VR simulation lesson to gain an indirect learning experience by watching the VR screen and other students taking part in the lesson. Students who completed the program were given 20 min to share and write about their opinions about the VR simulation, its strengths and weaknesses, areas of improvement, and when such a program should be operated if it is added to the curriculum. VR simulation took approximately 180 min to complete.

#### Debriefing

Upon completion of the VR simulation, the participants and instructor shared their experiences about the VR simulation for approximately 20 min, discussing similarity to reality, immersion, usefulness, and satisfaction. Moreover, feedback about areas of improvement was exchanged, and the students were given time to describe their thoughts.

### Data Collection Method

Data were collected from November 13 to December 10, 2021. Students participating in only the questionnaire survey (control group) and students participating in the VRICS program (experimental group) were recruited. Participants in the control and experimental groups had already completed the respiratory care for pediatric patients and the core basic nursing skill practice about donning and doffing standard PPE, not including level D PPE, and managing medical waste before this program. All participants completed an online preintervention survey. The control group completed an online postintervention survey 3 weeks after the preintervention survey. For preventing the diffusion of the experiment, the posttest for the control group was administered first. The experimental group completed a written postintervention survey after the completion of the program, and the completed questionnaire was placed in an envelope, which was retrieved by a research assistant. The survey required approximately 10-15 min to complete.

### Data Analysis

Collected data were analyzed using SPSS Statistics version 25.0 (IBM Corp). The general characteristics of the participants were expressed as real numbers and percentages, while homogeneity of the general characteristics between the experimental and control groups was tested using a chi-square test, the Fischer exact test, and an independent *t* test. The normal distribution of PPE knowledge, infection control performance, and self-efficacy scores was tested using the Shapiro-Wilk test. Normality test results indicated that the experimental group showed a normal distribution, but the control group did not show a normal distribution for PPE knowledge scores, and thus, a homogeneity test was performed using the Mann-Whitney test. Both experimental and control groups showed a normal distribution for infection control performance and self-efficacy scores, and thus, a homogeneity test was performed using an independent *t* test.

Differences in PPE knowledge between the experimental and control groups were tested using the Mann-Whitney *U* test and the Wilcoxon matched-pair signed-rank test, while differences in infection control performance and self-efficacy between the experimental and control groups were tested using an independent *t* test and a paired *t* test.

Realism of the program and satisfaction with the program in the experimental group were analyzed by the mean and SD.

### Ethical Considerations

After obtaining approval from the institutional review board of the institution affiliated with the researchers (GIRB-A21-Y-0061), an online preintervention survey was used to inform the participants about the objective of the study, their right to refuse participation during the study, and the no negative consequence on their school grade even if they did not participate in the study. Further, informed consent was obtained from the participants. Upon completion of the study, each participant was given a coffee gift certificate worth US $10-20.

## Results

### General Characteristics of the Participants and the Homogeneity Test

The general characteristics of the experimental and control groups and the PPE knowledge, infection control performance, and self-efficacy scores were homogeneous before the intervention (Table 2).

**Table 2:** Participants’ characteristics and homogeneity of the 2 groups.

Characteristics			Total (N=50)		Control group (N=25)		Experimental group (N=25)		*χ*^2^ (*df*)/*t* (*df*)/*z*		*P* value	
**College year, n (%)**									0			.99
	Third	26 (52.0)		13 (52.0)		13 (52.0)		N/A^a^		N/A		
	Fourth	24 (48.0)		12 (48.0)		12 (48.0)		N/A		N/A		
**Sex^b^, n (%)**									N/A			.35
	Male	5 (10.0)		1 (4.0)		4 (16.0)		N/A		N/A		
	Female	45 (90.0)		24 (96.0)		21 (84.0)		N/A		N/A		
**Age (years), n (%)**									2.03 (2)			.36
	20-21	18 (36.0)		9 (36.0)		9 (36.0)		N/A		N/A		
	22-23	18 (36.0)		11 (44.0)		7 (28.0)		N/A		N/A		
	≥24	14 (28.0)		5 (20.0)		9 (36.0)		N/A		N/A		
Age (years), mean (SD)			22.66 (2.08)		22.44 (2.02)		22.88 (2.15)		–0.75 (48)		.46	
**Previous semester grade^b^, n (%)**									2.17 (2)			.34
	<3.0	5 (10.0)		2 (8.0)		3 (12.0)		N/A		N/A		
	3.0-3.9	31 (62.0)		18 (72.0)		13 (52.0)		N/A		N/A		
	≥4.0	14 (28.0)		5 (20.0)		9 (36.0)		N/A		N/A		
**Experience of VR^c^, n (%)**									.32 (1)			.57
	Yes	24 (48.0)		11 (44.0)		13 (52.0)		N/A		N/A		
	No	26 (52.0)		14 (56.0)		12 (48.0)		N/A		N/A		
PPE^d^ knowledge, mean (SD)			14.86 (2.10)		14.52 (1.53)		15.20 (2.53)		–1.81		.07	
Infection control performance, mean (SD)			3.40 (0.69)		3.28 (0.65)		3.51 (0.71)		–1.18 (48)		.24	
Self-efficacy, mean (SD)			6.08 (0.64)		6.04 (0.60)		6.12 (0.68)		–0.42 (48)		.68	

^a^N/A: not applicable.

^b^Fisher exact test.

^c^VR: virtual reality.

^d^PPE: personal protective equipment.

### Testing of the Effects of the VRICS Program

The results of testing the effects of the VRICS program are shown in Table 3.

- Hypothesis 1 (difference in PPE knowledge): The experimental group showed a significant increase in the mean score from 14.52 points preintervention to 16.60 points postintervention (*z*=–3.85, *P*<.001). In contrast, the control group did not show a significant change in the mean score (*z*=–0.48, *P*=.63). The experimental group showed a significant increase in PPE knowledge compared to the control group as a result of the program (*z*=–3.28, *P*<.001). Thus, hypothesis 1 was accepted.
- Hypothesis 2 (difference in infection control performance): The experimental group showed a significant increase in the mean score from 3.28 points preintervention to 4.69 points postintervention (*t*_24_=6.47, *P*<.001). In contrast, the control group did not show a significant change in the mean score (*t*_24_=1.74, *P*=.095). The experimental group showed a significant increase in infection control performance compared to the control group as a result of the program (*t*_48_=4.89, *P*<.001). Thus, hypothesis 2 was accepted.
- Hypothesis 3 (difference in self-efficacy): The experimental group showed a significant increase in the mean score from 6.04 points preintervention to 6.64 points postintervention (*t*_24_=6.45, *P*<.001). Meanwhile, the control group showed a decrease in the mean score (*t*_24_=–2.20, *P*=.04). The experimental group showed a significant increase in self-efficacy compared to the control group as a result of the program (*t*_36.2_=4.93, *P*<.001). Thus, hypothesis 3 was accepted.

**Table 3:** Differences in variables between groups (N=50).

Group		Preintervention, mean (SD)	Postintervention, mean (SD)	Difference between time			Program effect					
				*t* (*df*)/*z*	*P* value	Postintervention-preintervention, mean (SD)		*t* (*df*)/*z*		*P* value		
**PPE^a^ knowledge**									–3.28^b^			<.001
	Experimental	14.52 (1.53)	16.60 (1.22)	–3.85^c^	<.001	2.08 (1.75)		N/A^d^		N/A		
	Control	15.20 (2.53)	15.52 (2.00)	–0.48^c^	.63	0.32 (2.08)		N/A		N/A		
**Infection control performance**									4.89 (48)			<.001
	Experimental	3.28 (0.65)	4.69 (0.82)	6.47 (24)	<.001	1.41 (1.09)		N/A		N/A		
	Control	3.51 (0.71)	3.71 (0.83)	1.74 (24)	.095	0.20 (0.58)		N/A		N/A		
**Self-efficacy**									4.93 (36.2)			<.001
	Experimental	6.04 (0.60)	6.64 (0.52)	6.45 (24)	<.001	0.60 (0.47)		N/A		N/A		
	Control	6.12 (0.68)	5.72 (0.79)	–2.20 (24)	.04	-0.40 (0.90)		N/A		N/A		

^a^PPE: personal protective equipment.

^b^Wilcoxon matched-pair signed-rank test.

^c^Mann-Whitney *U* test.

^d^N/A: not applicable.

### Realistic Immersion and Learner Satisfaction

The mean score for realistic immersion in the experimental group in the VR simulation experience was 4.49 (SD 0.50) points (out of 5 possible points), and the mean learner satisfaction score was 4.75 (SD 0.38) points (out of 5 possible points); see Table 4.

**Table 4:** Realistic immersion and learner satisfaction of the experimental group (n=25).

Items		Response, n(%)					Mean (SD)	
		Strongly disagree	Disagree	Neutral	Agree	Strongly agree		
**Realistic immersion**								
	1. The appearance of the VR simulation program video seemed to be real.	N/A^a^	N/A	N/A	11 (44.0)	14 (56.0)	4.56 (0.51)	
	2. While learning the VR simulation program, the screen seemed to exist in reality.	N/A	N/A	1 (4.0)	11 (44.0)	13 (52.0)	4.48 (0.59)	
	3. I felt like I was participating in a real field while learning the VR simulation program.	N/A	N/A	3 (12.0)	8 (32.0)	14 (56.0)	4.44 (0.71)	
	Overall	N/A	N/A	N/A	N/A	N/A	4.49 (0.50)	
**Learner satisfaction**								
	1. This program will help me work as a nurse in clinical practice.	N/A	N/A	N/A	9 (36.0)	16 (64.0)	4.64 (0.49)	
	2. This training is necessary as part of the nursing college curriculum.	N/A	N/A	N/A	5 (20.0)	20 (80.0)	4.80 (0.41)	
	3. I want to recommend this program to other nursing students.	N/A	N/A	N/A	5 (20.0)	20 (80.0)	4.80 (0.41)	
	Overall	N/A	N/A	N/A	N/A	N/A	4.75 (0.38)	

^a^N/A: not applicable.

## Discussion

### Principal Findings

In this quasi-experimental study, the cases of infection control for pediatric patients admitted to a negative-pressure isolation unit for COVID-19 infection were incorporated into a VR simulation program, and the effects of the program were assessed. Students who participated in the training program were third- and fourth-year nursing students with theoretical and clinical practice experience in pediatric nursing.

The findings of this study demonstrated that combining a VR simulation technique and skill training could enhance knowledge and skills for the preparation of respiratory infection care by future nurses. The experimental group, which participated in the VRICS program, showed significantly higher theoretical knowledge about PPE, infection control performance, and self-efficacy than the control group, which did not participate in the program. These findings suggest the need for VR simulation training before future nurses go to the front line to respond to respiratory infectious diseases. This VR simulation training was different than conventional training in that it provided simulated scenes of the isolation unit. Moreover, COVID-19 cases were incorporated into the training program. The order and accuracy of the donning and doffing of PPE and procedures related to respiratory care for pediatric patients were validated by infection control nurses and experts with at least 10 years of clinical experience in a neonatal intensive care unit (ICU) to simulate the actual environment related to COVID-19. Consequently, the participants were able to learn the procedure of donning PPE in the front zone and inspect the donned PPE before entering the isolation unit.

The participants were able to check the patient’s name and assess the respiratory status using lung sounds and patient monitoring values for the safety of the patient inside the isolation unit. Subsequently, the participants were able to learn the procedure for suctioning oral and nasal excretions from the pediatric patient, while preventing aerosol transmission. This step included procedures for accurately checking the suction pressure and excretion patterns. The participants also performed procedures for discarding suction tubes and disposable gloves used for respiratory care into the isolation unit medical waste container. They also performed the procedure of exiting the isolation unit and moving to the doffing room before accurately doffing the shoes, gown, gloves, goggles, and mask and discarding them in the waste container inside the contaminated zone. This program simulated the procedures involved in the assessment of clinical symptoms and nursing intervention for patients with COVID-19, contact isolation, and disinfection. The use of PPE can markedly reduce the infection risk associated with caring for patients with COVID-19 [33,34]. Therefore, accurate training for donning and doffing PPE is important.

With no signs of the COVID-19 pandemic subsiding worldwide, training for and acquiring of essential infection control skills are important for not only health care professionals but also for nursing students in coping with infectious diseases under such difficult circumstances. In the immersive VR learning environment using VRICS from this study, the participants can learn theories and skills essential for infection control with the incorporation of respiratory care for pediatric patients with COVID-19. Moreover, the VRICS program can reduce the risk of infection and ensure the safety of the trainees by avoiding direct contact with patients with COVID-19. VR simulation also offers the advantage of allowing individualized learning by overcoming the time and space constraints faced by traditional simulation learning during the COVID-19 pandemic, when gathering large groups is difficult.

A study by Zhang et al [3] developed a VR simulation training program and tested the effects of the program on the response capabilities of emergency reserve nurses facing a public health crisis. The results showed that there was a significant increase in emergency nursing–related knowledge, care ability, and disaster preparedness scores among nurses who participated in the program (*P*<.01). These results support the findings of our study showing a significant increase in PPE knowledge and infection control performance in the experimental group. However, technical ability increased significantly in the control group, and there was no difference in the postdisaster management score, which was contradictory to the findings of this study. Meanwhile, a study by Yu et al [4] reported that nursing students who participated in VR simulation programs for high-risk neonatal infection control showed greater improvement in self-efficacy for high-risk neonatal infection control performance (*t*_48_=–2.16, *P*=.02) than the control group that received only clinical practice training. These results support the findings of this study showing a significant difference in self-efficacy between the experimental and control groups.

Meanwhile, prebriefing was a crucial component of simulation learning [35,36], and the amount of information provided in prebriefing should be sufficient for the learner to begin problem solving [37]. It is believed that practicing donning and doffing PPE by VR simulation without individual practice or video-based learning would be difficult. Donning and doffing PPE is a complicated process that requires accuracy, and prior learning or practice is needed. Accordingly, this study used the concept of such prebriefing to carry out demonstrations and prior practice with donning and doffing PPE, along with an introduction to the scenarios. Previous studies have reported that improvement in satisfaction with simulation training [38], critical thinking disposition [39], immersion, and confidence [40] was achieved by prebriefing through watching a video that accurately presented the outcomes and goals. Moreover, when video-based prebriefing was applied, the self-efficacy of nursing students increased significantly in a team-based learning effect [41]. These common findings support the fact that video-based prebriefing further enhances the training effects of simulation compared to standard prebriefing that simply delivers information. According to Nayahangan et al [42], simulation-based training for medical staff has been mostly focused on clinical procedures related to practical skills. Ragazzoni et al [43] proposed a VR simulation model combining operational public health skills and hybrid skills training for infection control and Ebola treatment and management. Therefore, when using a VR simulation program, as in this study, diligently using prebriefing or applying a hybrid model could be a method for maximizing the learning effect.

According to Witmer and Singer [30], presence can be divided into 2 dimensions of involvement and immersion. In 3 dimensions, involvement can be further divided into spatial and temporal involvement and immersion can be further divided into dynamic and realistic immersion. Realistic immersion is defined as the perception of the environment that provides a realistic illusion. In this study, the experimental group showed a mean score of 4.49 (SD 0.50) points (out of 5 possible points) for the realistic immersion of VR simulation experience, which was relatively higher than the mean score of 2.75 (SD 0.91) points for realistic immersion reported by Chung [32], who developed the realistic immersion measurement tool. With respect to each item in the tool, “The appearance of the VR simulation program video seemed to be real” had the highest score (mean 4.56, SD 0.51 points), followed by “While learning the VR simulation program, the screen seemed to exist in reality” (mean 4.48, SD 0.59 points), and “I felt like I was participating in a real field while learning the VR simulation program” (mean 4.44, SD 0.71 points). The reason the realistic immersion for the screen seems to exist in reality, while learning and feel like participating in a real field showed relatively lower scores was because errors in sensor detection may have occurred due to differences in the heights of the participants or a change in the standing position may have caused the video to cut off as it was reloading. Moreover, immersion may have been reduced because the participants had to repeat the same motion several times due to poor recognition or had to return to the beginning due to the program being cut off. If the sensitivity of the sensor was increased and measures were taken to reduce program errors, then the participants may have been able to experience even high levels of realistic immersion.

The mean learner satisfaction score in this study was 4.75 (SD 0.38) points (out of 5 possible points), similar to 4.79 (SD 0.35) points reported in a study on high-risk neonatal VR simulation by Yu et al [4] and higher than 4.29 (SD 0.64) points reported in a study on general simulation training by Kim [44]. Among the items for learner satisfaction, “I want to recommend this program to other nursing students” and “This training is necessary as part of the nursing college curriculum” each showed a high mean score of 4.80 (SD 0.41) points (out of 5 possible points). It is believed that aspects of VR simulation that allowed the participants to use VR to indirectly experience the behavior in a neonatal ICU, where clinical training is difficult and can only be observed at the training site, and where practice can be repeated without the burden of committing mistakes, helped increase the level of learner satisfaction.

The participants showed a PPE knowledge score of 14-15 out of 20 points and an infection control performance score of 3 out of 5 points (average performance). Such results indicated that nursing students lacked PPE knowledge. Moreover, the control group, which did not participate in the VRICS program, showed a slight increase in yjr PPE knowledge score and no significant increase in infection control performance after 3 weeks. In the assessment of PPE knowledge using a questionnaire, the scores may have increased due to the participants remembering questions from the preintervention survey or being motivated to correct a wrong answer. No significant change in infection control performance in the control group indicates the need to provide programs or opportunities to teach skills that enhance COVID-19 infection control and allow the students to practice such skills.

Zellmer et al [45] reported that medical professionals did not remove their PPE properly during the Ebola virus outbreak, claiming that there is a need to improve the training that focuses on the protocol for properly wearing and removing PPE. Tabah et al [46] conducted an international study on PPE with 2711 ICU doctors and nurses during the COVID-19 pandemic. The results showed that N95 masks, full-sleeve waterproof gowns, and goggles were being washed or reused in 17%, 11%, and 34% cases, respectively, due to a shortage of PPE supplies. In fact, when conducting a prepractice in this study, level D PPE could not be individually provided to the participants due to the high cost and lack of supply during the COVID-19 pandemic. Accordingly, simulations such as VR offer an economically beneficial alternative to eliminating supply shortage. It is believed that in the future, simulation training can be integrated with a 3D virtual environment to optimize the training model so that the advantages of each feature can be maximized to enhance the overall clinical competency of nurses and nursing students.

### Limitations

This study had a few limitations. First, because the study population consisted of nursing students from a single college, caution should be taken when interpreting the results. Each college has different training programs, and students may have different levels of knowledge, performance, and self-efficacy, even if they are in the same grade. Second, the procedures for VR-based skills need to be revised to be more realistic. The VR program used in this study could not reproduce delicate procedures or nursing skills. This is because detailed procedures could not be introduced to each process, since the video run time was set to 15 min to prevent dizziness from wearing the HMD for too long, and allocation of the total operating time for the VR program. Moreover, because learning the skills for donning and doffing of PPE and respiratory care was a bigger goal, the program was set up to complete the applicable skill procedure just by hand touching through hand tracking. Therefore, when developing future VR programs, it is necessary to improve the imaging technology to reproduce even delicate motion. If not, learning effects could also be maximized by including the process of actually donning and doffing PPE, as in this study. The third limitation was testing of skills by means of a self-questionnaire in this study. Therefore, it is suggested that actual performance be evaluated using evaluation mode in a VR program.

### Conclusion

In this study, a VR simulation training program with the inclusion of infection control cases involving pediatric patients with COVID-19 admitted to an isolation unit was developed, and the effects of the program were tested. For infection control education, combining VR simulation and skills training was more effective than conventional instruction in producing better outcomes. This training program demonstrated more benefits for enhancing theoretical knowledge, respiratory care skills, and infectious disease preparedness, and thus, it could be applied to training nurses to respond even better to public health situations involving infectious diseases, including the COVID-19 pandemic.

## Abbreviations

HMD: head-mounted display
ICU: intensive care unit
PPE: personal protective equipment
VR: virtual reality
VRICS: virtual reality infection control simulation
